# Prediction of Ovarian Cancer-Related Metabolites Based on Graph Neural Network

**DOI:** 10.3389/fcell.2021.753221

**Published:** 2021-10-05

**Authors:** Jingjing Chen, Yingying Chen, Kefeng Sun, Yu Wang, Hui He, Lin Sun, Sifu Ha, Xiaoxiao Li, Yifei Ou, Xue Zhang, Yanli Bi

**Affiliations:** ^1^Department of Obstetrics and Gynecology, First Affiliated Hospital, Heilongjiang University of Chinese Medicine, Harbin, China; ^2^Department of Reproductive Medicine, Dalian Maternal and Children’s Centre, Dalian, China; ^3^Graduate School of Heilongjiang University of Chinese Medicine, Harbin, China; ^4^Department of General Practice, Beijing Friendship Hospital, Capital Medical University, Beijing, China; ^5^Department of Reproductive Medicine, The First Affiliated Hospital, Henan University of Chinese Medicine, Zhengzhou, China

**Keywords:** ovarian cancer, metabolite, Graph convolutional network, support vector machine, prediction

## Abstract

Ovarian cancer is one of the three most malignant tumors of the female reproductive system. At present, researchers do not know its pathogenesis, which makes the treatment effect unsatisfactory. Metabolomics is closely related to drug efficacy, safety evaluation, mechanism of action, and rational drug use. Therefore, identifying ovarian cancer-related metabolites could greatly help researchers understand the pathogenesis and develop treatment plans. However, the measurement of metabolites is inaccurate and greatly affects the environment, and biological experiment is time-consuming and costly. Therefore, researchers tend to use computational methods to identify disease-related metabolites in large scale. Since the hypothesis that similar diseases are related to similar metabolites is widely accepted, in this paper, we built both disease similarity network and metabolite similarity network and used graph convolutional network (GCN) to encode these networks. Then, support vector machine (SVM) was used to identify whether a metabolite is related to ovarian cancer. The experiment results show that the AUC and AUPR of our method are 0.92 and 0.81, respectively. Finally, we proposed an effective method to prioritize ovarian cancer-related metabolites in large scale.

## Introduction

Ovarian cancer is a common gynecological malignancy and one of the deadliest female diseases. Because the underlying symptoms are not obvious, about 70% of ovarian cancer patients are already at an advanced stage when they are diagnosed (Liu et al., 2020). The survival rates of patients with ovarian cancer at different stages are very different, and the mortality rate of patients with advanced stages exceeds 75% (Hussain, 2020). Therefore, there is an urgent need to find metabolites related to ovarian cancer to improve the prognosis of ovarian cancer and improve the efficiency of individualized treatment of patients (Perrone et al., 2020). Many life activities in cells occur at the metabolite level, so metabolomics has become one of the current research hotspots in the field of omics (Blimkie et al., 2020). The research of metabolomics in the early diagnosis of malignant tumors has shown its advantages (Agakidou et al., 2020). Ovarian cancer is a disease with a very high mortality rate of gynecological malignancies. There is an urgent need for a method to diagnose the disease early. The application of metabolomics in ovarian cancer can provide ideas for the diagnosis and prevention of ovarian cancer.

The analysis of the metabolites caused by the disease will help us to more comprehensively grasp the process of disease changes and the metabolic pathway of substances in the body, so as to make the clinical diagnosis more accurate. Zhou et al. (2010) collected 44 serous papillary ovarian cancer (stage I–IV) and 50 healthy women and found that histamine, purine nucleotide, glycine, serine, and sarcosine were the differential metabolites, and alanine, serine, cysteine, threonine, and glycine were overexpressed. Fong et al. (2011) found that there are 364 kinds of biochemical substances in human ovarian metabolic tissue by gas chromatography–mass spectrometry and liquid chromatography tandem mass spectrometry. Ovarian transformation can cause changes in energy utilization, resulting in glycolysis and fatty acids (such as carnitine, acetylcarnitine, and butyrylcarnitine) β-oxidation changes. Based on the non-targeted metabonomics method of LC/MS, Chen et al. (2011) analyzed the serum samples of 27 healthy women, 28 cases of benign ovarian tumor, and 29 cases of epithelial ovarian cancer. β-Cholestane-3,7,12,24,25, pentose glucoside, phenylalanine, glycine cholic acid, and propionyl carnitine are potential biomarkers for epithelial ovarian cancer.

Garcia et al. (2011) used the 1H NMR method to analyze the concentration of alanine, valine, phospholipid choline, etc., from the serum of 170 healthy women of appropriate age and 182 ovarian cancer stage I/II patients, while β-hydroxybutyrate, acetone, and acetoacetic acid have higher concentrations. These can be qualitatively compared with the changes in the concentration distribution of serum samples of cancer patients studied by other NMR-based metabolomics. This proves that early diagnosis of ovarian cancer can significantly affect the clinical outcome of patients with ovarian cancer. Chen et al. (2011) analyzed the serum samples of 27 healthy women, 28 cases of benign ovarian tumors, and 29 cases of epithelial ovarian cancer using LC/MS combined analysis, liquid chromatography selective ion monitoring mass spectrometry technology combined with PCR, and other pattern recognition techniques. The study found that 27-nor-5β-cholestane-3,7,12,24,25 pentanol glucuronide can be used in the early diagnosis of epithelial ovarian cancer. It is elevated in the serum of early epithelial ovarian cancer (stage I). Gaul et al. (2015) used ultra-high performance liquid chromatography and high-resolution mass spectrometry from 46 early (I/II) serous epithelial ovarian cancer (EOC) patients and 49 age-matched normal healthy female controls. UPLC-MS and tandem mass spectrometry (MS/MS) methods found that 16 metabolites in lipids and fatty acids have 100% accuracy in the diagnosis of early-stage ovarian cancer patients. Woo et al. (2009) tested the metabolites in urine of 10 breast cancer patients, 9 ovarian cancer patients, 12 cervical cancer patients, and 22 normal controls. They found that 1-Methyladenosine is a powerful biomarker for diagnosing ovarian cancer. Zhang et al. (2012) found that 2-Piperidinone could be used to distinguish epithelial ovarian cancer (EOC) and benign ovarian tumor (BOT).

Although multiple metabolites have been found to be related to ovarian cancer, the time and money cost of this discovery is huge. With the development of computational method, increasing number of researchers try to use machine learning or deep learning methods to solve biological problems (Chen et al., 2019, 2020; Zhao et al., 2020b). Disease-related genes (Peng and Zhao, 2020; Zhao et al., 2021), RNAs (Gebauer et al., 2021), proteins (Zhao et al., 2020c), and drugs (Tianyi et al., 2020) have all been identified by computational methods in large scale, which significantly increases the speed of discovering knowledge. Predicting disease-related metabolites by computational methods has become a hot issue in recent years. Hu et al. (2018) used random walk to identify disease-related metabolites by similarity network in 2018. Following this research, Wang et al. (2019) fused text mining technology with random walk to further infer relationship between metabolites and diseases. Then, Peng and Zhao (2020) developed “MDBIRW,” which is an improved random walk method to identify disease-related metabolites. However, these methods all traverse network by random walk, which did not fully extract the topological relationship of similarity network. In 2020, Zhao et al. (2020a) proposed “Deep-DRM,” which used Graph convolutional network (GCN) to encode similarity network and achieved high accuracy. In this paper, we followed this research and focused on ovarian cancer to provide support for the treatment and diagnosis of ovarian cancer by prioritizing metabolites.

## Materials and Methods

**FIGURE 1:**
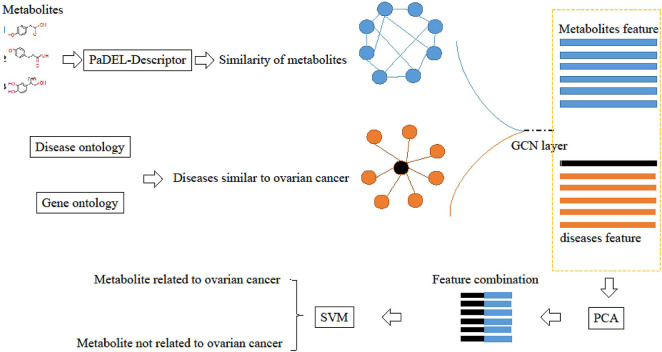
Workflow of GPS-OCM (the fusion of GCN, PCA, and SVM to identify ovarian cancer-related metabolites).

Deep-DRM is a method that fuses GCN, principal component analysis (PCA), and deep neural network (DNN). Considering we only focus on ovarian cancer, the sample set would be much smaller. Therefore, we used support vector machine (SVM) to replace DNN to build a model with a small sample. The workflow of our method is shown in Figure 1.

### Metabolite and Disease Similarity Network

We used “PaDEL-Descriptor” (Yap, 2011) to estimate the chemical property of metabolites by their chemical structure. The output of this tool includes 1D and 2D descriptors and fingerprints. Each metabolite could be represented as a vector of 2,325 dimension in this way.


(1) $$m_{i}=\left[v_{1},v_{2},\ldots .,v_{2325}\right]$$

The similarity of each of the two metabolites could be calculated by vector cosine.


(2) $$s\,i\,m\,\left({\hat{m}}_{i},{\hat{m}}_{j}\right)=\frac{\sum_{k}^{2325}{\hat{m}}_{i}^{k}\times {\hat{m}}_{j}^{k}}{\sqrt{\sum_{k}^{2325}{\left({\hat{m}}_{i}^{k}\right)}^{2}}\times \sqrt{\sum_{k}^{2325}{\left({\hat{m}}_{j}^{k}\right)}^{2}}}$$

Using the similarities of metabolites, we could build a metabolite similarity network. In the network, each node is a metabolite and each edge is the similarity between the two metabolites.

Cheng et al. (2014) proposed SemFunsim to calculate disease similarity. We used their results to build an ovarian cancer similarity network. All the nodes in this network are diseases similar to ovarian cancer. The edges are the similarities between ovarian cancer and other diseases.

### Feature Encoding by Graph Convolutional Network

Graph convolutional network was implemented on both metabolite and disease similarity networks, respectively. The GCN-based network feature extraction method can convert the network structure into a vector output through a non-linear function:


(3) $$H^{\left(l+1\right)}=f\,\left(H^{\left(l\right)},A\right)$$

*H*^(0)^ = *X* which is the initial feature of each node.

First, we need to perform Laplacian changes on the network, and the corresponding Laplacian matrix calculation formula is as follows:


(4) L=D-A

Among them, D is the degree matrix of the graph, which can be solved by formula 5. A is the adjacency matrix.


(5) $${\hat{\text{D}}}_{\text{ii}}=\sum_{j}{\hat{\text{A}}}_{i\,j}$$

Since D is a diagonal matrix, only its diagonal elements need to be solved, and the remaining elements are all 0.

Then, we need to normalize the Laplacian matrix:


(6) $$L^{s\,y\,m}=D^{-\frac{1}{2}}\,L\,D^{-\frac{1}{2}}=I-D^{-\frac{1}{2}}\,A\,D^{-\frac{1}{2}}$$

The final formula of GCN would be:


(7) $$H^{\left(l+1\right)}=\sigma \,\left(D^{-\frac{1}{2}}\,A\,D^{-\frac{1}{2}}\,H^{\left(l\right)}\,W^{\left(l\right)}\right)$$

σ() is the activation function, *W*^(*l*)^ the parameter to be trained.

Finally, we obtained the encoded feature of metabolites and ovarian cancer.

### Feature Dimensionality Reduction

Since the dimension of metabolites and ovarian cancer features are large, we used PCA to reduce the dimension.

Principal component analysis reduces the n-dimensional input data to r-dimensional, where r < n. PCA is essentially a basis transformation, so that the transformed data have the largest variance, that is, by rotating the coordinate axis and translating the origin of the coordinate, the variance between one of the axes (main axis) and the data point is minimized. After the coordinate conversion, the orthogonal axis with high variance is removed, and the dimensionality reduction data set is obtained.

The SVD method is used to perform PCA dimensionality reduction. Assuming that there are p × n-dimensional data samples X, there are p samples in total, and each row is n-dimensional. The p × n real matrix can be decomposed into:


(8) $$X=U\,\sum V^{T}$$

Here, the dimension of the orthogonal matrix U is p × n, the dimension of the orthogonal matrix V is n × n (orthogonal matrix satisfies: *UU^T^* = *VV^T^* = 1), and Σ is a diagonal matrix of n × n. Next, divide Σ into r columns, denoted as Σr; use U and V to get the dimensionality reduction data point Yr:


(9) $$Y_{r}=U\,\sum_{r}$$

After PCA, 99% of the feature information are preserved for both metabolites and ovarian cancer.

### Identify Ovarian Cancer-Related Metabolites

After extracting and reducing the features of metabolites and ovarian cancer, we need to combine features of metabolites and ovarian cancer to make ovarian cancer–metabolite pairs. If the metabolite has relationship with ovarian cancer, the label of this pair would be 1; otherwise, the label is 0.

**FIGURE 2:**
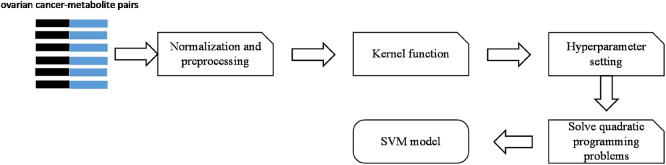
Process of building the SVM model.

There are five steps to build the SVM model. The process is shown in Figure 2.

## Experiment Results

Since we only focus on ovarian cancer, we divided our experiments into two classes. One is to identify ovarian cancer-related metabolites from known disease-related metabolites, which is named as “SP.” The other one is to identify ovarian cancer-related metabolites from metabolites associated with no disease, which is names as “SM”.

**FIGURE 3:**
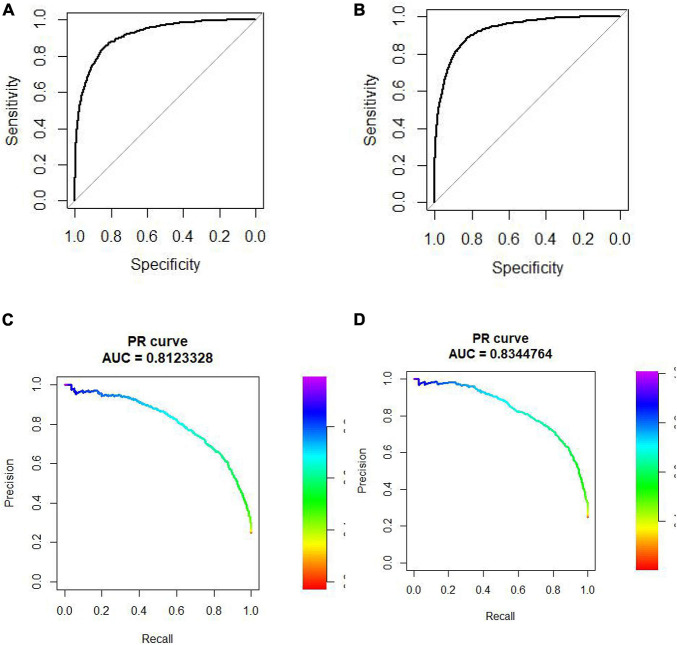
**(A)** ROC curve of “SP.” Experiment. **(B)** ROC curve of “SM” experiment. **(C)** PR curve of “SP” experiment. **(D)** PR curve of “SM” experiment.

We did 10-cross validation on both “SP” and “SM” experiments. The AUC and AUPR of these experiments are shown in Figure 3.

The AUC of “SP” and “SM” experiments is 0.9168 and 0.9282, respectively. The AUPR of “SP” and “SM” experiments is 0.81 and 0.83, respectively.

To show the superiority of GPS-OCM, we compared GPS-OCM with RWPS-OCM, GPR-OCM, and GPD-OCM. We replaced GCN by Random Walk (RW) to construct RWPS-OCM. GPR-OCM is the fusion of GCN, PCR, and Random Forest (RF). GPD-OCM is to replace SVM by deep neural network (DNN). The results are shown in Table 1. GPS-OCM performed best among these methods.

**TABLE 1:** Comparison experiment of GPS-OCM and other methods on the “SP” test.

**Method**	**AUC**	**AUPR**
GPS-OCM	0.92	0.81
RWPS-OCM	0.83	0.70
GPR-OCM	0.87	0.73
GPD-OCM	0.90	0.81

After verifying the effectiveness of our method, we used all known ovarian cancer-related metabolites as positive samples and randomly selected equal number of other metabolites as negative samples to build a final GPS-OCM model. We totally identified 257 more metabolites that are associated with ovarian cancer. To verify whether these metabolites are associated with ovarian cancer, we chose the top five of these metabolites and did case studies.

Three of the top five metabolites have been reported to be related to ovarian cancers. Niemi et al. (2017) used morning urine samples from 23 women with benign ovarian tumors and 37 women with malignant ovarian tumors and found that N1, N12-Diacetylspermine showed significant statistical differences, and found that it can help distinguish benign and malignant ovarian tumors as well as early and advanced stage, and low malignant potential and high-grade ovarian cancers from each other, respectively. Fahrmann et al. (2021) found that 3-acetamidopropyl can significantly increase the sensitivity of ovarian cancer diagnosis by 116 ovarian cancer patients and 143 controls. Dessources et al. (2017) collected samples from 16 patients with benign ovarian pathology and 21 patients with malignant pathology and found that multiple metabolites are significantly associated with ovarian cancer including N-acetylation, acyl carnitines, and tryptophan.

## Conclusion

Identifying ovarian cancer-related metabolites can help better understand pathogenic mechanism and disease process. In addition, metabolites in blood and urine have shown strong power in diagnosing cancer in early stage as biomarkers. However, few metabolites associated with ovarian cancer have been found at present. In order to speed up the study of metabolites related to ovarian cancer, we proposed a calculation method “GPS-OCM” based on similarity of metabolites and diseases. This method is fusion of GCN, PCA, and SVM. GCN was used to extract network topology features and PCA was implemented to reduce the dimension of disease and metabolite features. SVM was applied to do classification. The experiments show the high accuracy of our method with high AUC and AUPR. In addition, three of the top five metabolites that are identified as ovarian cancer-related metabolites by our method have been proven by previous studies, which proved the accuracy of our results.

## Data Availability Statement

The datasets presented in this study can be found in online repositories. The names of the repository/repositories and accession number(s) can be found in the article/supplementary material.

## Author Contributions

JC and YC wrote this manuscript. YW and HH did experiments. KS, SH, XL, and YO contributed to software analysis. LS provided important ideas. XZ and YB guided the whole work. All authors contributed to the article and approved the submitted version.

## Conflict of Interest

The authors declare that the research was conducted in the absence of any commercial or financial relationships that could be construed as a potential conflict of interest.

## Publisher’s Note

All claims expressed in this article are solely those of the authors and do not necessarily represent those of their affiliated organizations, or those of the publisher, the editors and the reviewers. Any product that may be evaluated in this article, or claim that may be made by its manufacturer, is not guaranteed or endorsed by the publisher.
